# WTAP Suppresses Cutaneous Melanoma Progression by Upregulation of KLF9: Insights into m6A-Mediated Epitranscriptomic Regulation

**DOI:** 10.3390/biomedicines13112685

**Published:** 2025-10-31

**Authors:** Huayu Huang, Dong Li, Yichuan Li, Ying Wang, Jin Yin

**Affiliations:** 1 Department of Dermatology, Tongji Hospital, Tongji Medical College, Huazhong University of Science and Technology, Wuhan 430030, China; h1187048191@gmail.com (H.H.); lidong_2022@hust.edu.cn (D.L.); liyichuan0217@163.com (Y.L.); wangggwin@163.com (Y.W.); 2 Department of Hematology, Tongji Hospital, Tongji Medical College, Huazhong University of Science and Technology, Wuhan 430030, China

**Keywords:** m6A methylation, cutaneous melanoma, epigenetic regulation, WTAP, KLF9

## Abstract

**Background**: N6-methyladenosine (m6A) modification plays a crucial role in tumor biology; however, the function of the methyltransferase adaptor WTAP in melanoma remains poorly understood. **Methods**: We analyzed WTAP expression and its clinical relevance using TCGA-SKCM and GTEx datasets, followed by immunohistochemical validation in melanoma tissues. The biological effects of WTAP were assessed through gain- and loss-of-function experiments in melanoma cell lines. Weighted gene co-expression network analysis (WGCNA) and LASSO regression were used to identify key WTAP-related genes. **Results**: WTAP expression was significantly decreased in melanoma compared with normal skin and was negatively correlated with tumor progression and poor survival. Functionally, WTAP overexpression suppressed melanoma cell proliferation and migration, whereas its knockdown produced the opposite effects. Bioinformatic analyses and rescue experiments identified KLF9 as a potential downstream effector of WTAP. WTAP depletion reduced KLF9 mRNA and protein levels, while overexpression restored them. Moreover, MeRIP-qPCR confirmed that WTAP promotes m6A enrichment on KLF9 mRNA, suggesting a post-transcriptional regulatory mechanism. **Conclusions**: Our findings reveal a novel WTAP–KLF9 axis that mediates melanoma suppression through m6A-dependent regulation. This study provides new insight into the context-specific role of WTAP in melanoma and suggests it may serve as a potential biomarker or therapeutic target.

## 1. Introduction

Arising from dysregulated growth and malignant conversion of melanocytes, cutaneous melanoma represents a highly aggressive skin tumor [1]. While cutaneous melanoma is a rare type of skin cancer, it exhibits high levels of aggressiveness and mortality. The main subtypes of cutaneous melanoma are superficial spreading melanoma, nodular melanoma, malignant lentigo melanoma, and acral lentiginous melanoma [2]. Melanocytes derive from a structure in vertebrate embryos known as the neural crest, and their primary function is melanin production, which is a crucial pigment that plays a significant role in protecting the skin from sunlight exposure and neutralizing reactive oxygen species [3]. In recent decades, the incidence of cutaneous melanoma has rapidly increased worldwide [4]. According to the latest GLOBOCAN 2022 report, there were approximately 331,647 new cases of cutaneous melanoma and 58,645 deaths worldwide, with the global incidence rate showing a steady upward trend over the past 20 years [5]. Multiple treatment strategies are accessible for cutaneous melanoma, with the optimal choice influenced by factors such as the tumor’s anatomical site, stage, genetic features, as well as the patient’s age and general health condition. In cases of intermediate to advanced stages, a comprehensive treatment approach is often preferred [6]. Despite advances in immunotherapy and targeted therapy, 5-year survival for distant melanoma (cancer has metastasized) remains about 34.6% [7]. Given the poor prognosis of advanced melanoma and the limited efficacy of current treatments, identifying novel molecular targets is crucial for improving patient outcomes.

m6A, known as N6-methyladenosine, is the most prevalent RNA modification in eukaryotic cells [8]. While m6A was first discovered in the 1970s, its exact functions and mechanisms have only been largely understood recently. m6A modification is essential for regulating different stages of RNA metabolism, including processing, degradation, nuclear export, and translation, thus precisely calibrating RNA expression and function [9]. The dynamic and reversible nature of m6A RNA methylation in mammals is similar to that of DNA and histone methylation, suggesting it as a potential new layer of epigenetic regulation [10]. The genes involved in the m6A process are classified into three categories [11,12,13]: “writers”, “erasers” and “readers”, encoding proteins capable of adding, removing, or recognizing m6A modification sites and thereby modulating crucial biological processes. The “writers”, or methyltransferases—including METTL3, METTL14, METTL16, ZC3H13, WTAP, and KIAA1429—catalyze the addition of m6A marks to RNA. In contrast, the “erasers” such as FTO and ALKBH5 function as demethylases, reversing these modifications. The “readers”, comprising m6A-binding proteins like YTHDF1/2/3, YTHDC1/2, IGF2BP3, and HNRNPA2B1, recognize and interpret m6A marks to mediate downstream effects. Notably, several writer and reader proteins, including METTL3, WTAP, METTL16, and ZC3H13, can assemble into methyltransferase complexes [14]. These complexes, in coordination with erasers, contribute to the dynamic regulation and homeostasis of m6A methylation.

The regulatory mechanism of m6A methylation is complex. In recent years, studies have shown that m6A modification plays a vital role in various biological processes, particularly drawing extensive attention for its regulatory mechanisms in tumor initiation and progression. For example, reducing the expression of METTL3 or METTL14 has been shown to increase glioblastoma cell proliferation and self-renewal, while elevated levels of METTL3 have inhibitory effects [15]. However, in acute myeloid leukemia, upregulation of METTL3 and METTL14 is crucial for the self-renewal and maintenance of leukemia stem cells [16]. FTO is upregulated in various cancers, including cervical cancer, breast cancer, and acute myeloid leukemia [17,18,19]. High levels of WTAP expression have been observed in osteosarcoma, gastric cancer, and cholangiocarcinoma [20,21,22], while it is downregulated in bladder urothelial carcinoma in a pan-cancer research [23]. Multiple m6A genes have been extensively studied in various tumor fields, with some already serving as targets for disease monitoring and treatment. However, limited published research on m6A genes in cutaneous melanoma remains. Therefore, it is necessary to explore further the potential connection between m6A genes and cutaneous melanoma.

**Figure 1:**
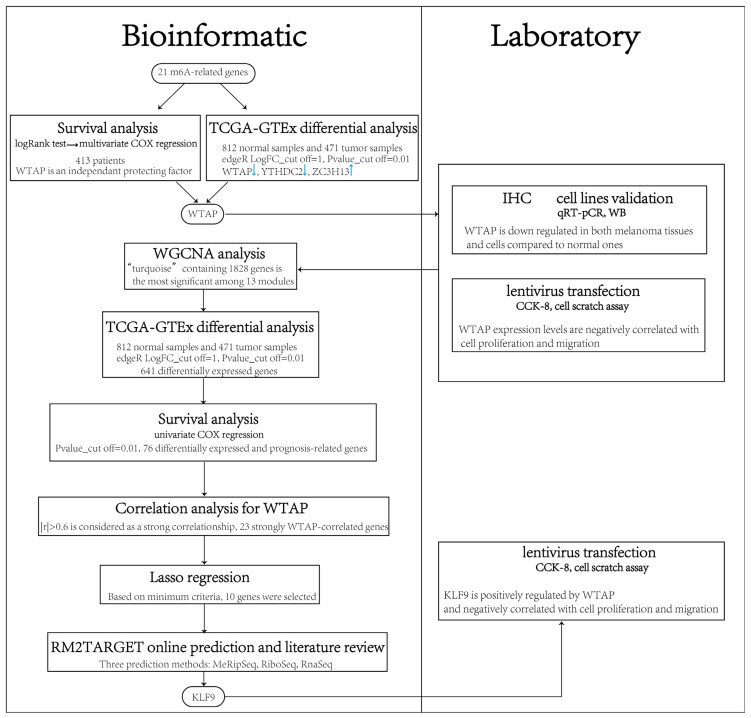
Workflow for identifying differentially expressed and prognostically relevant m6A regulators and their downstream targets, along with the potential mechanisms underlying their roles in cutaneous melanoma.

We conducted an in-depth analysis of m6A regulatory factors, emphasizing WTAP, to evaluate their expression, clinical prognostic value, and functional impact in cutaneous melanoma (Figure 1). These insights may contribute to advancing m6A-based strategies for melanoma diagnosis and treatment.

## 2. Materials and Methods

### 2.1. Gene Expression Data

From the TCGA database, we accessed RNA sequencing data along with clinical survival information, encompassing 471 melanoma cases and a single normal tissue control. Considering the limited number of normal skin tissue samples in TCGA-SKCM, an additional 811 normal skin RNA-seq datasets were retrieved from the GTEx database to supplement the control group. Probe identifiers were mapped to corresponding official gene symbols based on platform annotations. When multiple probes corresponded to the same gene, the one with the highest expression value was retained. The raw expression matrix was then normalized using the log2 (x + 1) transformation.

### 2.2. Selection of m6A Genes

To broaden the scope of this study, 21 classic m6A genes were selected (ALKBH5, CBLL1, ELAVL1, FMR1, FTO, HNRNPA2B1, HNRNPC, IGF2BP1, KIAA1429, LRPPRC, METTL14, METTL3, RBM15, RBM15B, WTAP, YTHDC1, YTHDC2, YTHDF1, YTHDF2, YTHDF3, ZC3H13), representing a large proportion of currently well-studied “readers”, “writers”, and “erasers” in the field of oncology.

### 2.3. Differential Analysis

Differential expression analysis of the selected 21 m6A-related genes in cutaneous melanoma was performed using the “edgeR” package in R. Visualization of the results was performed through heatmaps and violin plots. Subsequently, the same R package (version 4.4.1) and dataset were employed to screen for potential target genes associated with m6A genes. Differential expression was determined for genes meeting the criteria of logFC > 1 or <−1 and *p*-value < 0.01.

### 2.4. Survival Analysis

Considering potential sample duplications and missing clinical data in cutaneous melanoma samples, a cohort of 413 patients with both RNA transcription details and complete survival records was identified in the TCGA database. Utilizing the above dataset, multivariable Cox regression analysis was sequentially employed to pinpoint genes linked to the survival outcomes of cutaneous melanoma patients among the selected 21 m6A genes. Subsequently, genes related to WTAP and patient survival were identified through repeated application of univariate Cox analysis and Lasso regression.

### 2.5. Immunohistochemical Staining

90 cutaneous melanoma samples and 11 normal skin tissue samples were obtained from Tongji Hospital, affiliated with Tongji Medical College of Huazhong University of Science and Technology. Immunohistochemical staining was performed using tissue microarrays. This study was approved by the Institutional Review Board of Tongji Hospital (No. TJ-IRB20220174). The rabbit anti-WTAP primary antibody (Cusabio, Cat# CSB-PA618970LA01HU, Wuhan, China) was at a 1/200 dilution. Semi-quantitative analysis was conducted by measuring the average optical density (AOD) of the DAB signal using ImageJ software (version 1.54g) (National Institutes of Health, Bethesda, MD, USA). For each sample, at least five randomly selected fields were captured under identical light intensity and exposure settings. The AOD was defined as the mean gray value of the positively stained area, representing the relative expression intensity of the target protein. All analyses were conducted in a blinded manner by two independent pathologists, and the average of their measurements was used for statistical comparison.

### 2.6. Cell Culture and Transfection

Human melanoma cell lines A375, A2058, MNT-1, and normal melanocyte line PIG-1 were obtained from the Cell Bank of the Chinese Academy of Sciences (Shanghai, China). Cells were cultured in Dulbecco’s Modified Eagle Medium (DMEM, Cat# 10566016, Gibco) supplemented with 10% heat-inactivated certified fetal bovine serum (FBS, Cat# A5670701, Gibco, Thermo Fisher Scientific, Waltham, MA, USA), 100 U/mL penicillin, and 100 µg/mL streptomycin. Expression constructs for WTAP and KLF9 were generated using the plvx-ires-puro vector, and shRNAs targeting these genes or a control sequence were inserted into the pLKO.1-puro vector. The lentiviral particles were collected from the culture medium, purified through centrifugation and ultrafiltration, and stored at −80 °C. A375 and A2058 cells were seeded in 6-well plates and allowed to grow until reaching approximately 40% cell density. An appropriate amount of lentiviral particles was added to the culture medium based on the desired multiplicity of infection (MOI). After 24 h, cells were refreshed with 10% FBS-containing medium and incubated for another 48 h. The transfection efficiency of WTAP and KLF9 was verified by Western blot and qRT-PCR.

### 2.7. RNA Isolation and Quantitative Real-Time PCR

Total RNA was extracted using the Super FastPure Cell RNA Isolation Kit (Vazyme, Cat# RC-102-01, Nanjing, China) according to the manufacturer’s instructions. Real-time PCR analysis was performed using the SYBR Green PCR mix (TOYOBO, Cat# QPS-201,Osaka, Japan) on a CFX Connect Real-Time PCR Detection System (Bio-Rad, Hercules, CA, USA). Comparative quantitative mRNA levels of cells were normalized to the housekeeping gene GAPDH. Primers for real-time PCR were synthesized in Tsingke Biotechnology (Beijing, China).

### 2.8. Quantification of mRNA m6A Methylation by MeRIP-qPCR

To determine the effect of WTAP on the m6A modification of KLF9 mRNA, m6A RNA immunoprecipitation followed by quantitative PCR (MeRIP-qPCR) was performed using the RiboMeRIP m6A Transcriptome Profiling Kit (Ribobio, Cat# C11051-1, Guangzhou, China) according to the manufacturer’s instructions. A375 cells were transduced with either sh-NC or sh-WTAP lentivirus prior to total RNA extraction. Briefly, 5 μg of purified and fragmented mRNA was incubated with 5 μg of anti-m6A antibody or magnetic A/G beads alone in 500 μL of IP buffer for 2 h at 4 °C with gentle rotation. The methylated RNA-antibody complexes were eluted using free m6A and subsequently purified with the HiPure Serum miRNA Kit (Magen, Cat# R431402, Guangzhou, China). One-tenth of the fragmented RNA was reserved as input for normalization. The relative enrichment of m6A on KLF9 mRNA in each sample was quantified by RT-qPCR and expressed as the ratio of MeRIP to input RNA.

### 2.9. Western Blotting

Cells were collected and washed three times with PBS. Cell samples were lysed using a combination of RIPA, a protease inhibitor, and a phosphatase inhibitor. Protein concentration normalization was conducted using loading buffer and RIPA lysis buffer. Proteins were first subjected to separation using SDS-polyacrylamide gel electrophoresis and then immobilized onto a PVDF membrane. To prevent non-specific binding, membranes were blocked in 5% BSA for 1 h and then incubated overnight at 4 °C with the appropriate primary antibodies. After washing, the membrane is incubated with the secondary antibody labeled with horseradish peroxidase (Invitrogen, Thermo Fisher Scientific, Waltham, MA, USA).

### 2.10. Cell Viability Assay

Cell viability was assessed using the Cell Counting Kit-8 (CCK-8), obtained from Yeasen Biotechnology (Cat# 40203ES60, Shanghai, China). Cells were inoculated into a 96-well plate at a density of 3 × 10^3^ cells/well and cultured overnight. To investigate the relationship between WTAP expression and cell viability, cells were treated with the well-constructed lentivirus mentioned above for 72 h. After discarding the supernatant, each well received 10 µL CCK-8 solution and 100 µL Medium, followed by incubation at 37 °C in the dark. Subsequently, the OD values at 450 nm were obtained using a microplate reader after incubating for 90 min and 120 min.

### 2.11. Cell Scratch Assay

During the exponential growth phase, A375 and A2058 cells were treated with 0.25% trypsin for digestion. The resulting cell suspension was diluted to 2.5 × 10^5^ cells/mL in complete DMEM and plated in 6-well plates (5 × 10^5^ cells/well). When the cells formed a full monolayer at the bottom of the well, the medium was removed, and the cells were rinsed three times with DMEM. A 10 μL pipette tip was then used to create several linear scratches, and non-adherent cells were eliminated by washing with PBS 2–3 times. Cells were subsequently maintained in a CO_2_ incubator, and wound healing was monitored by photographing the scratch area at baseline and 24 h post-incubation.

### 2.12. Weighted Gene Correlation Network Analysis and LASSO Regression Analysis

We used the “WGCNA” package to identify potential pathways involved in WTAP-mediated regulation of melanoma proliferation and migration. In the TCGA-SKCM dataset, the top 10% of genes (n = 5839) was ranked by median absolute deviation (MAD) and selected in descending order. Gene modules were constructed based on WTAP expression and patients’ clinical traits. Correlations were computed between module eigengenes and the aforementioned clinical or molecular traits to identify strongly associated modules.

LASSO regression was then performed using the “glmnet” R package to identify key WTAP-related genes from the turquoise module obtained by WGCNA. A 10-fold cross-validation approach was employed to select the optimal λ value that minimized the mean cross-validated error. Genes with non-zero coefficients at this λ value were considered hub genes. To ensure robustness, the procedure was repeated 1000 times with random resampling, and only genes appearing in more than 80% of the iterations were retained for downstream analysis.

### 2.13. Correlation Analysis

To identify genes potentially associated with WTAP, Pearson correlation analysis was conducted between WTAP and the 76 genes identified in the prior upstream analysis, based on RNA expression data from all tumor and normal samples (n = 1283). Genes with correlation coefficients > 0.6 or <−0.6 and a *p*-value < 0.01 were considered as WTAP-related genes.

### 2.14. WTAP Targets Online Prediction

To predict WTAP-regulated genes, we used RM2Target, a database integrating targets of RNA modification-related WERs across nine epigenetic marks, including m6A. The predictive results are derived from three sources: MeRIP-Seq, Ribo-Seq, and RNA-Seq. The intersection of these sources is considered the final candidate gene set for WTAP target gene prediction.

### 2.15. Statistical Analysis

Normally distributed quantitative data are presented as mean ± standard deviation (SD), whereas skewed data are expressed as median (25th–75th percentile). Categorical variables are shown as frequency (percentage). Statistical inference was performed using appropriate tests according to data distribution and variance homogeneity. Specifically, comparisons between two groups were analyzed using an unpaired two-tailed Student’s *t*-test, and multiple-group comparisons were conducted using one-way ANOVA followed by Tukey’s post hoc test.

All functional assays, including CCK-8 cell proliferation, cell scratch, and rescue experiments, were independently repeated at least three times. Data are expressed as mean ± SD from biological replicates. Differences were considered statistically significant at *p* < 0.05. All statistical analyses and data visualizations were performed using R software (version 4.4.1) and GraphPad Prism (version 9.5.1).

## 3. Results

### 3.1. Differential Expression of m6A Genes in Cutaneous Melanoma

**Figure 2:**
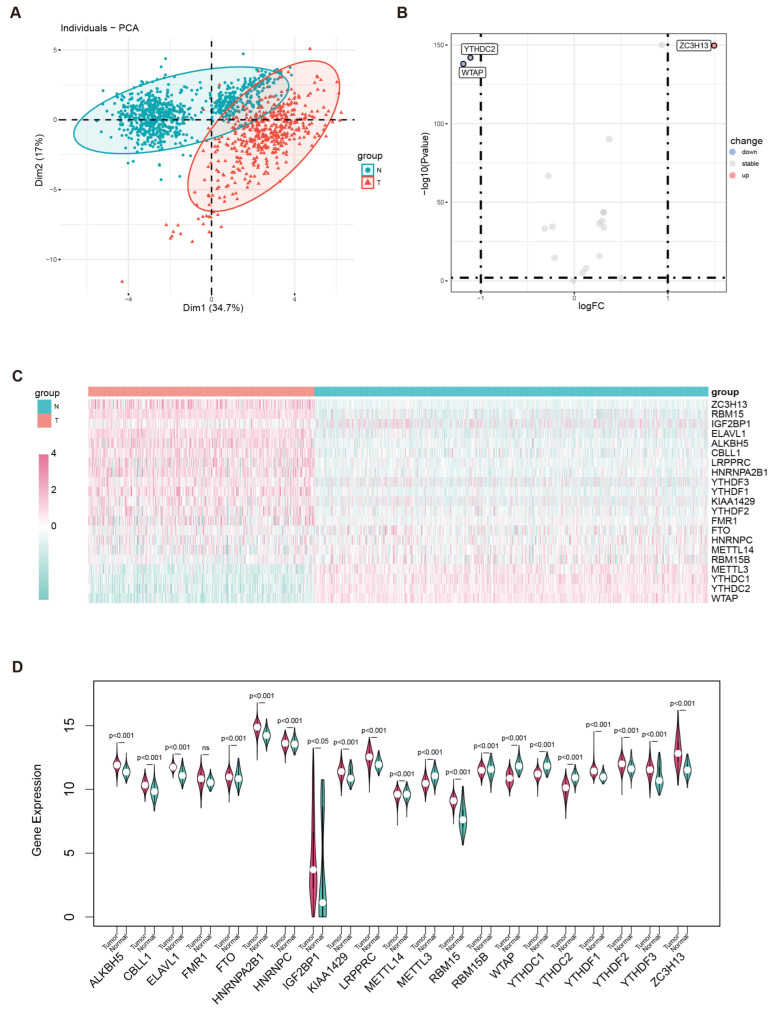
**Identification of m6A gene expression differences based on the merged data from TCGA-SKCM and GTEx-SKIN.** (**A**) Principal components analysis of the whole genome between normal skin and cutaneous melanoma. (**B**) Volcano map of 21 m6A genes in the aforementioned dataset. (**C**) Heatmap illustrating the differential expression patterns of 21 m6A-related genes. (**D**) Violin plot displaying the expression levels of 21 m6A-related genes in normal skin and cutaneous melanoma samples.

The gene expression dataset for cutaneous melanoma included 471 tumor samples (all derived from TCGA-SKCM) and 812 normal skin samples, comprising 811 from GTEx-SKIN and one from TCGA-SKCM. The expression of 21 m6A genes was extracted and compared between melanoma and normal samples based on the integrated datasets. Principal components analysis showed a relatively different distribution pattern between normal and tumor groups (Figure 2A). Compared to normal samples, WTAP and YTHDC2 were downregulated, whereas ZC3H13 expression was elevated in tumor tissues among the 21 genes examined (Figure 2B). Expression differences were depicted using both a heatmap (Figure 2C) and a violin plot (Figure 2D).

### 3.2. Survival Analysis of m6A Genes in Cutaneous Melanoma

**Figure 3:**
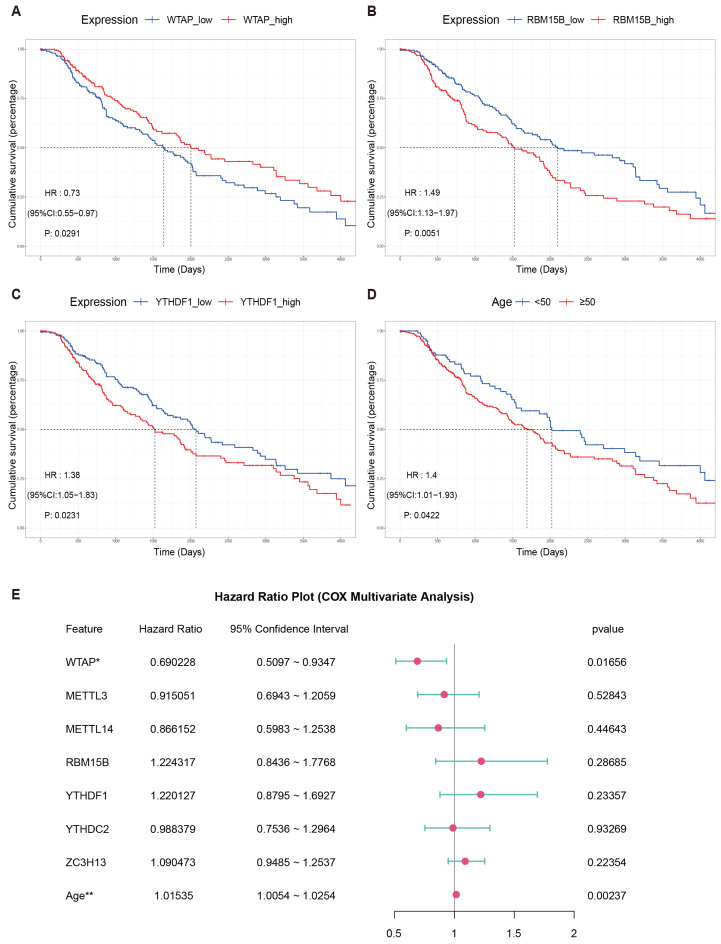
**Prognostic analysis of m6A genes.** (**A**–**D**) Kaplan–Meier plots showing the survival impact of WTAP, RBM15B, YTHDF1, and age in 413 melanoma patients from TCGA-SKCM. (**E**) Forest plot from the multivariate Cox model includes WTAP, METTL3, METTL14, RBM15B, YTHDF1, YTHDC2, ZC3H13 and age. * *p* < 0.05, ** *p* < 0.01.

Prognostic information from 413 TCGA-SKCM tumor cases was used to perform survival analysis on the 21 m6A genes. WTAP showed a protective effect on prognosis, with higher expression associated with better survival outcomes (HR: 0.73, 95% CI: 0.55–0.97, *p* = 0.029). In contrast, increased expression of RBM15B (HR: 1.49, 95% CI: 1.13–1.97, *p* = 0.005) and YTHDF1 (HR: 1.38, 95% CI: 1.05–1.83, *p* = 0.023) predicted worse clinical outcomes (Figure 3A–C).

Considering the differential expression of YTHDC2 and ZC3H13, as well as the close relationship between METTL3 and METTL14 with WTAP, along with the association of age with prognosis (Figure 3D), these factors were collectively utilized for a multivariate survival analysis. The forest plot results confirmed WTAP as an independent prognostic gene (HR: 0.69, 95% CI: 0.51–0.93, *p*: 0.01656 < 0.05) (Figure 3E).

### 3.3. Verification of WTAP Downregulation and Investigation of Its Association with Melanoma Cells Properties

**Figure 4:**
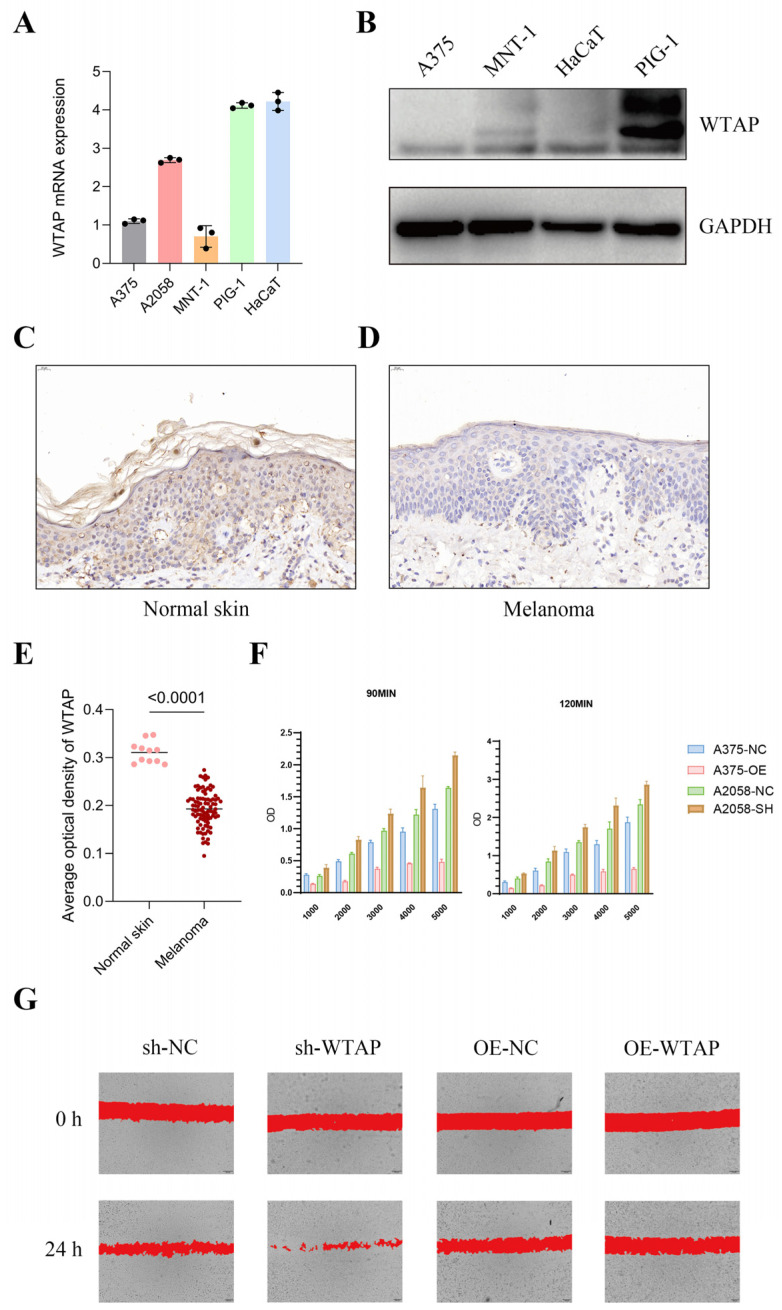
Verification of WTAP downregulation and investigation of its association with melanoma cell proliferation and migration. (**A**) WTAP mRNA levels in cell lines. (**B**) WTAP protein levels in cell lines with GAPDH as the endogenous control. (**C**,**D**) WTAP expression status in tissues. (**E**) Average optical density of WTAP in tissues. (**F**) OD450 values of A375 cells and A2058 cells after lentivirus transfection. (**G**) Cell migration ability of A375 cells and A2058 cells after lentivirus transfection. All data are presented as mean ± SD from three independent experiments. Statistical significance was determined using Student’s *t*-test. *p* < 0.05 was considered significant.

WTAP expression was significantly decreased in melanoma cell lines (A375, A2058, MNT-1) compared with normal melanocytes (PIG-1), as confirmed both in RNA and protein levels (Figure 4A,B). A similar result was also seen in cutaneous melanoma tissues (Figure 4C–E). Additionally, the basal expression level of WTAP appeared to be positively associated with the proliferative and migratory capacities of melanoma cell lines (A375 and A2058) (Figure S1A,B).

To explore the functional role of WTAP in cutaneous melanoma, lentiviral transfection was performed in A375 and A2058 cells to induce either overexpression or knockdown of WTAP. The efficiency of transfection was confirmed using qRT-PCR. Overexpression of WTAP led to a significant reduction in melanoma cell proliferation, while its silencing increased proliferative activity (Figure 4F and Figure S1C,D). The migration ability of melanoma cells was parallel to the proliferation ability when facing WTAP overexpression or knockdown (Figure 4G).

### 3.4. Correlation Between Modules and Phenotypes in WGCNA

**Figure 5:**
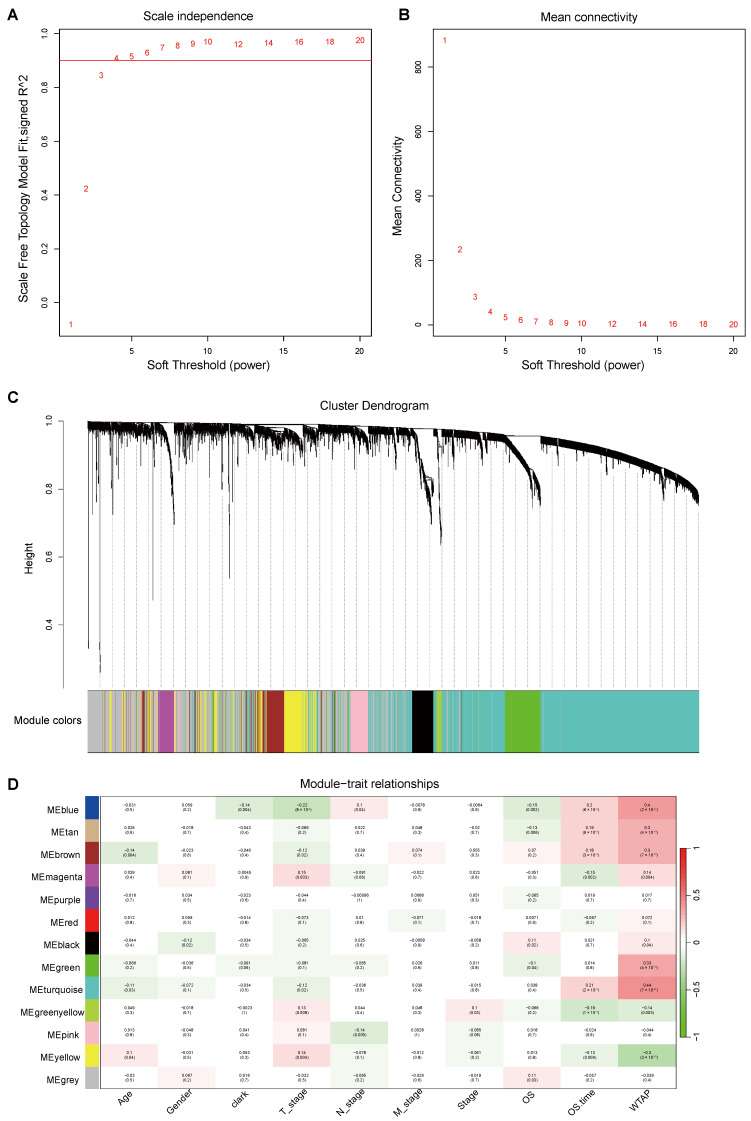
**WGCNA module detection and their correlation with phenotypes, especially WTAP.** (**A**,**B**) The scale-free topology index and average connectivity were evaluated under various soft-thresholding powers, with β = 4 chosen. (**C**) Hierarchical clustering resulted in 13 color-coded modules based on topological overlap. (**D**) Heatmap showing correlations between module eigengenes and phenotypic traits, including WTAP; correlation coefficients and *p*-values are indicated outside and inside the brackets, respectively.

For co-expression network analysis, genes were ranked by MAD calculated from 413 melanoma samples in TCGA-SKCM, and the top 5839 genes (top 10%) were selected. The soft-thresholding power β was determined to be 4 to construct a scale-free co-expression network while preserving reasonable mean connectivity across gene nodes (Figure 5A,B). This led to the detection of 13 gene modules (Figure 5C).

On the basis of module–trait relationships, those modules demonstrating strong correlations with WTAP expression were identified for further analysis (Figure 5D). The turquoise module, showing the highest correlation with WTAP expression (r = 0.44, *p* = 7 × 10^−21^), was prioritized for downstream analysis. The positive coefficient implies a direct association with WTAP, and its high statistical significance highlights a strong connection to patient prognosis in cutaneous melanoma. These results suggest that WTAP may influence the expression of genes within this module, thereby impacting disease outcomes.

### 3.5. Differential Analysis and Survival Analysis of Module Genes and Selection of Potential Target of WTAP

**Figure 6:**
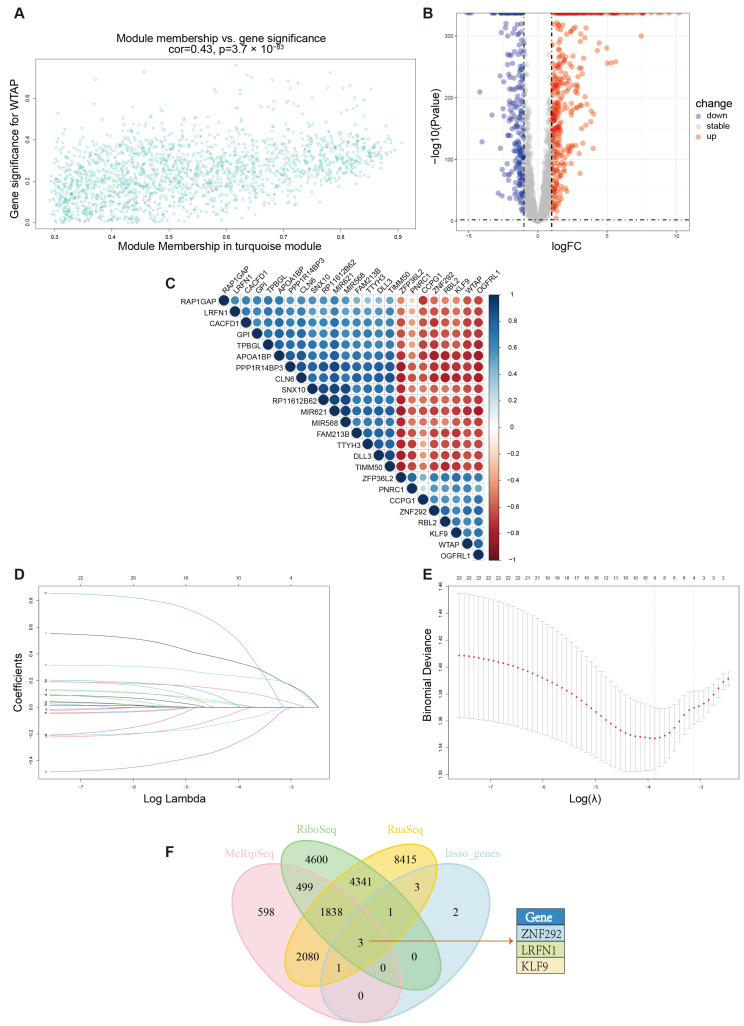
**Selection of potential targets of WTAP in module genes.** (**A**) The scatter diagram of correlation between turquoise module genes (n = 1818) and WTAP expression. (**B**) Visualization of gene expression changes in the turquoise module using a volcano plot derived from TCGA-GTEx data. (**C**) The correlation among 23 strongly WTAP-correlated genes and WTAP. (**D**,**E**) Determination of the number of factors by the LASSO analysis. (**F**) Venn map of RM2Target online prediction.

The turquoise module showed a positive correlation with WTAP (r = 0.43, *p* < 0.001; Figure 6A). From this module, 1818 genes were selected for subsequent analysis to identify WTAP-associated prognostic markers. Differential expression analysis using combined TCGA-SKCM and GTEx-SKIN data excluded 1177 non-differential genes, leaving 641 differentially expressed ones (410 upregulated and 231 downregulated; Figure 6B).

Among the 641 differentially expressed module genes, 76 were found to be prognostically relevant through univariable Cox regression analysis (Table S1). Correlation analysis further selected 23 strongly WTAP-correlated genes from the above 76 genes with an absolute value of r > 0.6 (Figure 6C). A LASSO regression model was constructed using TCGA-SKCM expression data of the 23 selected genes to refine further prognostic marker identification (Figure 6D,E). Moreover, 10 hub genes in the risk model were used for prediction, potentially binding to and regulated by WTAP (Table S2).

To further refine potential WTAP targets, we consulted the RM2Target database, which integrates MeRIP-Seq, Ribo-Seq, and RNA-Seq datasets to predict targets of m6A-related proteins. By intersecting these predicted genes with the LASSO-selected hub genes, we identified three overlapping candidates (Figure 6F). Among them, KLF9 was ultimately selected for functional validation based on several lines of supporting evidence.

Supplementary expression analysis revealed that KLF9 and IFNGR1—among the LASSO-selected genes—were consistently downregulated in melanoma samples compared to normal skin across multiple independent datasets, including TCGA and several GEO cohorts (Figure S2A). This expression pattern mirrored that of WTAP.

High expression of both KLF9 and IFNGR1 was significantly linked to favorable overall survival outcomes in melanoma, as demonstrated by Kaplan–Meier analysis (Figure S2B), indicating their possible protective roles similar to WTAP.

Analysis using the JASPAR database identified two high-affinity KLF9 binding motifs within the WTAP promoter region, suggesting that KLF9 may transcriptionally regulate WTAP, potentially forming a feedback loop (Figure S2C).

Given its consistent downregulation, prognostic relevance, and potential regulatory relationship with WTAP, KLF9 was prioritized for subsequent functional validation experiments.

### 3.6. WTAP Regulates Cell Proliferation and Migration in Melanoma Cells Through KLF9

**Figure 7:**
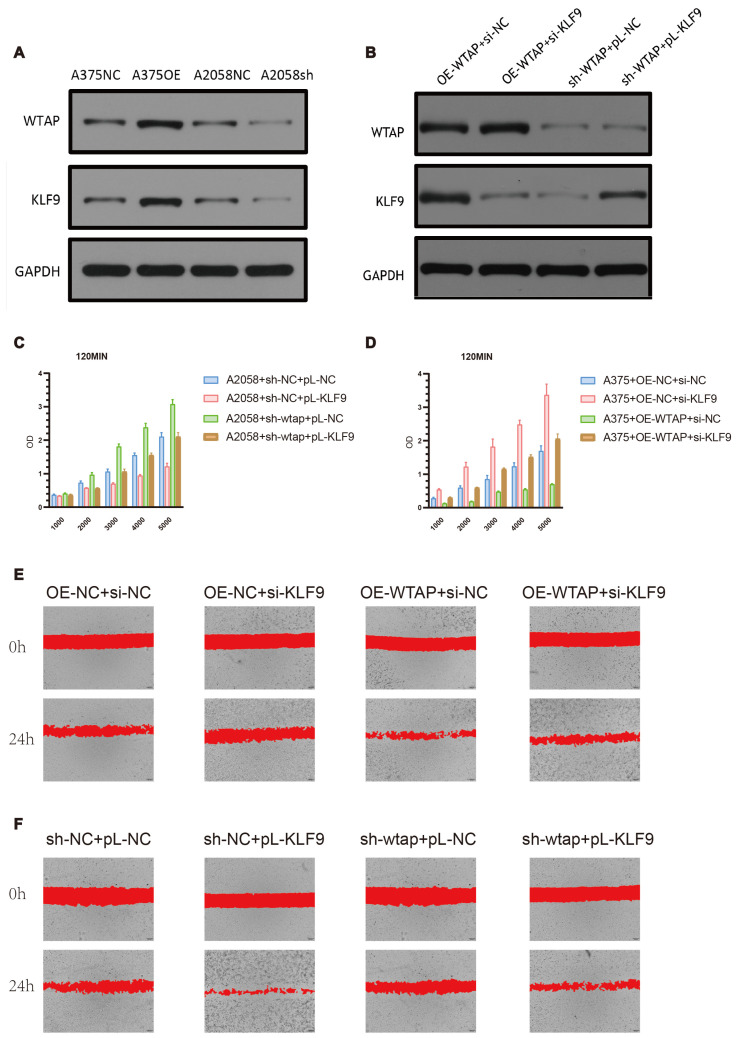
**WTAP regulates cell proliferation and migration in melanoma cells through KLF9.** (**A**,**B**) Expression levels of WTAP and KLF9 in melanoma cells after lentivirus transfection. (**C**,**D**) Proliferation ability of cells after lentivirus transfection. (**E**,**F**) Migration ability of cells after lentivirus transfection. All data are presented as mean ± SD from three independent experiments. Statistical significance was determined using one-way ANOVA with Tukey’s post hoc test. *p* < 0.05 was considered significant.

To investigate the relationship between WTAP and KLF9, we utilized lentivirus transfection to overexpress or knock down WTAP or KLF9. WTAP was demonstrated to be capable of positively regulating expression levels of KLF9 (Figure 7A,B). Importantly, rescue experiments confirmed the functional interaction between WTAP and KLF9: the suppressive effects of WTAP overexpression were partially reversed by KLF9 knockdown, and conversely, the pro-proliferative effects of WTAP knockdown were mitigated by KLF9 overexpression (Figure 7C,D). The migration ability of melanoma cells was parallel to cell proliferation when facing WTAP/KLF9 overexpression or knockdown (Figure 7E,F).

To further verify whether WTAP modulates KLF9 expression through an m6A-dependent mechanism, we performed MeRIP-qPCR in A375 cells. The results showed that silencing WTAP significantly reduced the m6A enrichment level of KLF9 mRNA compared with the sh-NC group (*p* < 0.0001), indicating that WTAP is required for the m6A methylation of KLF9 transcripts (Figure S3).

## 4. Discussion

Cutaneous melanoma accounts for approximately 3% of malignant skin tumors [24], characterized by its high malignancy, rapid growth, and pronounced propensity for metastasis, often leading to diagnosis at advanced stages. Current treatment strategies for such patients primarily involve extensive surgical resection accompanied by adjuvant therapies including chemotherapy, immunotherapy, targeted therapy, and other approaches [25]. However, the efficacy of these treatments remains suboptimal due to melanoma’s resistance to targeted therapy and limited response to immunotherapy. For instance, despite advances in immunotherapy, the 5-year survival rate for patients with distant metastatic melanoma remains below 40%. This highlights the urgent need to identify novel molecular mechanisms and therapeutic targets to improve patient prognosis.

Given the extensive research and potential applications of m6A genes in various tumor fields, we focused on melanoma. Based on published studies, a total of 2 eraser genes, 3 writer genes, and 5 reader genes have been reported to play a role in melanoma. For eraser genes, studies have found that knocking down FTO in mouse models could increase the tumor’s sensitivity to PD-1 therapy [26], while ALKBH5 promoted melanoma cell proliferation, migration, and invasion by acting on FOXM1 [27] and ABCA1 [28]. Additionally, YTHDF2 [29] and YTHDF3 [30,31] facilitated melanoma progression through distinct mechanisms. Regarding writer genes, analysis of the TCGA database revealed that RBM15B was an independent protective prognostic gene for uveal melanoma [32]. On the contrary, multiple reports have indicated that METTL3 [33,34,35,36,37,38,39] and METTL14 [40,41] were risky and drug-resistant genes associated with melanoma. Collectively, these findings underscore the diverse and context-dependent roles of m6A regulators in melanoma. However, many key regulators, such as WTAP, remain poorly characterized in cutaneous melanoma and warrant further investigation.

In order to systematically investigate the role of m6A in melanoma, we selected a total of 21 widely studied m6A genes, including the aforementioned 8 genes. WTAP was found to be downregulated in cutaneous melanoma based on TCGA and GTEx data, and its higher expression correlated with more favorable patient outcomes. Utilizing these databases, Feng [42] and Li [43] also identified WTAP as a protective prognostic gene. Subsequently, we confirmed the downregulation of WTAP in melanoma at both the tissue and cellular levels and further discovered a negative correlation between WTAP expression levels and the proliferative and migratory capabilities of melanoma cells.

As a binding partner of Wilms’ tumor 1, WTAP is involved in multiple cellular functions, such as alternative splicing, regulation of cell proliferation, and cell cycle control [44,45,46]. As a classic m6A writer and a component of the methyltransferase complex, the primary function of WTAP is to methylate target RNA. Through further exploration of public databases, we identified KLF9 as a potential target gene through which WTAP exerts its effects in melanoma. KLF9, a zinc finger protein involved in transcriptional regulation, is found in a wide range of human tissues, including the skin, brain, lung, kidney, and intestine [47]. Several antioxidant genes, including TXNRD2, are inhibited by KLF9, thereby increasing reactive ROS levels, which negatively impact tumor development [48]. KLF9 has been found to be downregulated in various human cancers, and its overexpression can inhibit several tumorigenic phenotypes in tumor cells, such as proliferation, invasion, and anti-apoptosis [49,50,51]. Similarly to other tumors, KLF9 also plays a protective role in melanoma. Bagati et al. [52] found that KLF9 deficiency promoted melanoma proliferation and metastasis in mouse models. Altonsy et al. [53] discovered that upregulation of KLF9 expression enhanced the sensitivity of RPMI-7951 and A375 melanoma cell lines to the chemotherapeutic agent paclitaxel. Our findings revealed that KLF9 was downregulated in cutaneous melanoma, and its higher expression was associated with improved clinical prognosis. Subsequently, in cell experiments, we demonstrated that KLF9 was positively regulated by WTAP. As anticipated, KLF9 also showed a negative correlation with the proliferative and migratory capabilities of melanoma cells.

Recent research has emphasized the significance of m6A RNA methylation regulators in melanoma, particularly the role of WTAP. WTAP was recently identified through bioinformatics analysis as a potential prognostic biomarker in cutaneous melanoma, characterized by lower expression in tumor samples and a positive association with overall survival. Our findings are consistent with this report, as we also observed downregulation of WTAP in melanoma tissues and cell lines, along with a positive correlation between WTAP expression and overall survival.

However, our study extends beyond prognostic association and provides mechanistic insights into how WTAP may exert its tumor-suppressive effects. Specifically, we identified KLF9, a known tumor suppressor, as a potential downstream effector of WTAP. Functional rescue experiments confirmed that WTAP inhibits melanoma cell proliferation and migration at least in part through upregulating KLF9. Given that KLF9 itself has been implicated in restraining malignant phenotypes in various cancers, including melanoma, this WTAP-KLF9 axis offers a novel mechanistic link between m6A regulatory machinery and melanoma suppression.

To further substantiate the proposed m6A-mediated mechanism, we performed MeRIP-qPCR to assess whether WTAP regulates the methylation of KLF9 mRNA directly. The results showed a significant decrease in m6A enrichment on KLF9 transcripts upon WTAP knockdown in melanoma cells, suggesting that WTAP is required for maintaining the m6A modification of KLF9. Together with the observation that WTAP overexpression increased KLF9 mRNA and protein levels, these findings provide the first direct experimental evidence that WTAP positively regulates KLF9 expression through an m6A-dependent epitranscriptomic mechanism. This strengthens our hypothesis that the tumor-suppressive function of WTAP in melanoma is, at least in part, mediated by its role as an m6A “writer” on KLF9 mRNA.

While our data demonstrate that WTAP enhances KLF9 expression and increases m6A enrichment on KLF9 mRNA, the precise mechanism—whether purely post-transcriptional or also involving effects on protein stability—remains to be elucidated. WTAP may regulate KLF9 translation efficiency or protein turnover indirectly through m6A-“reader” proteins such as IGF2BPs or HuR, which have been reported to stabilize and protect target transcripts from degradation. Future work using protein stability assays and ribosome profiling will be required to distinguish between these transcriptional and post-transcriptional effects.

Although our rescue experiments demonstrated that KLF9 overexpression counteracted the oncogenic effects of WTAP depletion, KLF9 is a multifunctional transcription factor that may regulate melanoma progression through WTAP-independent pathways. Therefore, while the current data support a WTAP–KLF9 regulatory link, we cannot exclude the possibility that KLF9 also exerts separate tumor-suppressive functions. Future transcriptomic profiling and chromatin-binding analyses will help distinguish the overlapping and independent effects of these two regulators.

While previous studies provided preliminary evidence for WTAP’s prognostic value, our work not only confirms its protective role but also reveals a new functional pathway through which WTAP may limit melanoma progression. These findings contribute to a more comprehensive understanding of WTAP’s role in melanoma biology and may open avenues for developing targeted therapeutic strategies aimed at modulating the WTAP/KLF9 pathway.

Interestingly, although WTAP, METTL3, and METTL14 are core components of the same m6A methyltransferase complex, they do not necessarily exert identical biological effects across cancers. Several reports indicate that WTAP can also function independently of METTL3/METTL14 through its interactions with RNA-binding proteins, thereby influencing RNA stability, splicing, or localization in a target-specific manner [44,45]. In melanoma, this context-specific regulation may explain why WTAP displays tumor-suppressive properties, in contrast to the oncogenic roles of METTL3 and METTL14. Furthermore, the outcome of m6A modification is known to depend on the relative activity of m6A “readers” and “erasers,” suggesting that WTAP might preferentially enhance m6A modification on transcripts such as KLF9 that contribute to tumor inhibition. These findings underscore the complexity and context dependency of m6A-mediated regulation in melanoma and highlight the need for further investigation into the specific cofactor interactions that determine the directionality of WTAP function.

Although m6A “writer” complexes are often associated with promoting mRNA decay via YTHDF2-dependent pathways, accumulating evidence indicates that the outcome of m6A modification is highly context-dependent [54,55]. WTAP-mediated m6A marks can also enhance mRNA stability or translation when recognized by “reader” proteins such as IGF2BP1/2/3 or YTHDF1, which bind m6A-modified transcripts and protect them from degradation. WTAP has additionally been reported to interact with HuR (ELAVL1) and HNRNPC, which further contributes to the stabilization of its target mRNAs [44]. Therefore, the observed upregulation of KLF9 in response to WTAP may result from m6A-dependent stabilization or enhanced translation mediated by these reader complexes. This interpretation highlights the dynamic nature of m6A regulation and provides a mechanistic explanation for the unconventional WTAP–KLF9 axis observed in melanoma.

However, this study has certain limitations. For example, we did not find differential expression of some genes that have been previously studied, such as the METTL and YTHDF gene series, and other m6A genes when utilizing TCGA and GTEx databases, which may suggest a potential bias in the public information. Additionally, the present study systematically elucidated the WTAP–KLF9 regulatory axis and its m6A-dependent mechanism in melanoma cells, all findings were based on in vitro experiments. The lack of in vivo validation limits the direct translational interpretation of our results. Future studies employing melanoma xenograft or transgenic mouse models are warranted to confirm the physiological relevance of the WTAP–KLF9 pathway and to explore its potential as a therapeutic target in vivo. Most importantly, among numerous studies on m6A genes, it is noteworthy that WTAP exhibits a close association with METTL3 and METTL14, collectively forming a methyltransferase complex, which suggests a synergistic relationship among these three genes. Nonetheless, METTL3 and METTL14 are risk genes in melanoma based on published research, while the results of this study indicated WTAP as a protective factor. More intriguingly, whether as a writer like METTL3 and METTL14 or as an eraser like FTO and ALKBH5, all these are tumor promoting genes in melanoma, implying that the balance between methylation and demethylation in the tumor field needs to be precisely assessed, not only at an overall level but also accurately targeted to each specific gene. This complexity underscores the need for continuous exploration and excavation in the future.

## 5. Conclusions

In summary, our findings reveal that WTAP is a downregulated m6A regulator in cutaneous melanoma and plays a tumor-suppressive role by restraining melanoma cell proliferation and migration. Importantly, we reveal a novel WTAP–KLF9 regulatory axis, in which WTAP positively modulates the expression of the tumor suppressor gene KLF9, thereby contributing to its anti-tumor function. These findings expand the current understanding of the epitranscriptomic landscape in melanoma and provide mechanistic insights that may inform the development of targeted m6A-based therapeutic strategies. The m6A-related regulatory pathways require deeper investigation, and in vivo validation will be essential to support our current findings.

## Data Availability

The data presented in this study are openly available in TCGA-SKCM and GTEx database.

## Supplementary Materials

The following supporting information can be downloaded at: https://www.mdpi.com/article/10.3390/biomedicines13112685/s1, Figure S1. WTAP inhibits melanoma cell proliferation and migration in vitro. (A) Celltrace proliferation assay showing baseline proliferative activity in melanoma cell lines A375 and A2058 and normal melanocytes MNT-1. (B) Wound healing assay in A375 and A2058 cells. (C) CCK-8 proliferation assay in A375 and A2058 cells after WTAP overexpression (OE) or knockdown (SH). (D) EdU incorporation assay to assess DNA synthesis. Quantitative analyses of migration distances and EdU-positive cells are presented as mean ± SD (n = 3, *p* < 0.05). Scale bar = 100 μm. OE, overexpression; SH, short hairpin RNA knockdown; EdU, 5-ethynyl-2′-deoxyuridine; CCK-8, Cell Counting Kit-8; SD, standard deviation; Figure S2: Identification of KLF9 as a WTAP-associated gene with prognostic significance in melanoma. (A) Heatmap illustrating the correlation of candidate gene expression across TCGA-SKCM and multiple GEO datasets (GSE3189, GSE4587, GSE56494, GSE112509). (B) Kaplan–Meier survival curves for melanoma patients based on gene expression levels of IFNGR1 (left) and KLF9 (right). (C) Predicted KLF9-binding sites in the WTAP promoter region, based on transcription factor motif analysis using the JASPAR database; Figure S3. WTAP promotes m6A methylation enrichment of KLF9 mRNA. MeRIP-qPCR analysis showing that knockdown of WTAP significantly reduced m6A enrichment on KLF9 transcripts in A375 cells compared with the sh-NC group. Data are presented as mean ± SD of three independent experiments, by unpaired Student’s *t*-test; Table S1: Prognostic genes identified by univariable Cox regression among differentially expressed candidates from the WTAP-associated module; Table S2: Ten hub genes identified by LASSO regression as potential WTAP-associated prognostic markers in cutaneous melanoma.

## Abbreviations

The following abbreviations are used in this manuscript:

WTAP	Wilms’ tumor 1-associating protein
KLF9	Krueppel-like factor 9
m6A	N6-methyladenosine
TCGA	The Cancer Genome Atlas
GTEx	Genotype-Tissue Expression
LASSO	Least absolute shrinkage and selection operator
AOD	Average optical density
MOI	Multiplicity of infection
CCK-8	Cell counting kit-8
WGCNA	Weighted gene correlation network analysis
HR	Hazard ratio
CI	Confidence interval
