# Supplementary figures and images for: WTAP Suppresses Cutaneous Melanoma Progression by Upregulation of KLF9: Insights into m6A-Mediated Epitranscriptomic Regulation
Source: Biomedicines. 2025 Oct 31;13(11):2685. doi: 10.3390/biomedicines13112685 (PMC12650262; doi:10.3390/biomedicines13112685)

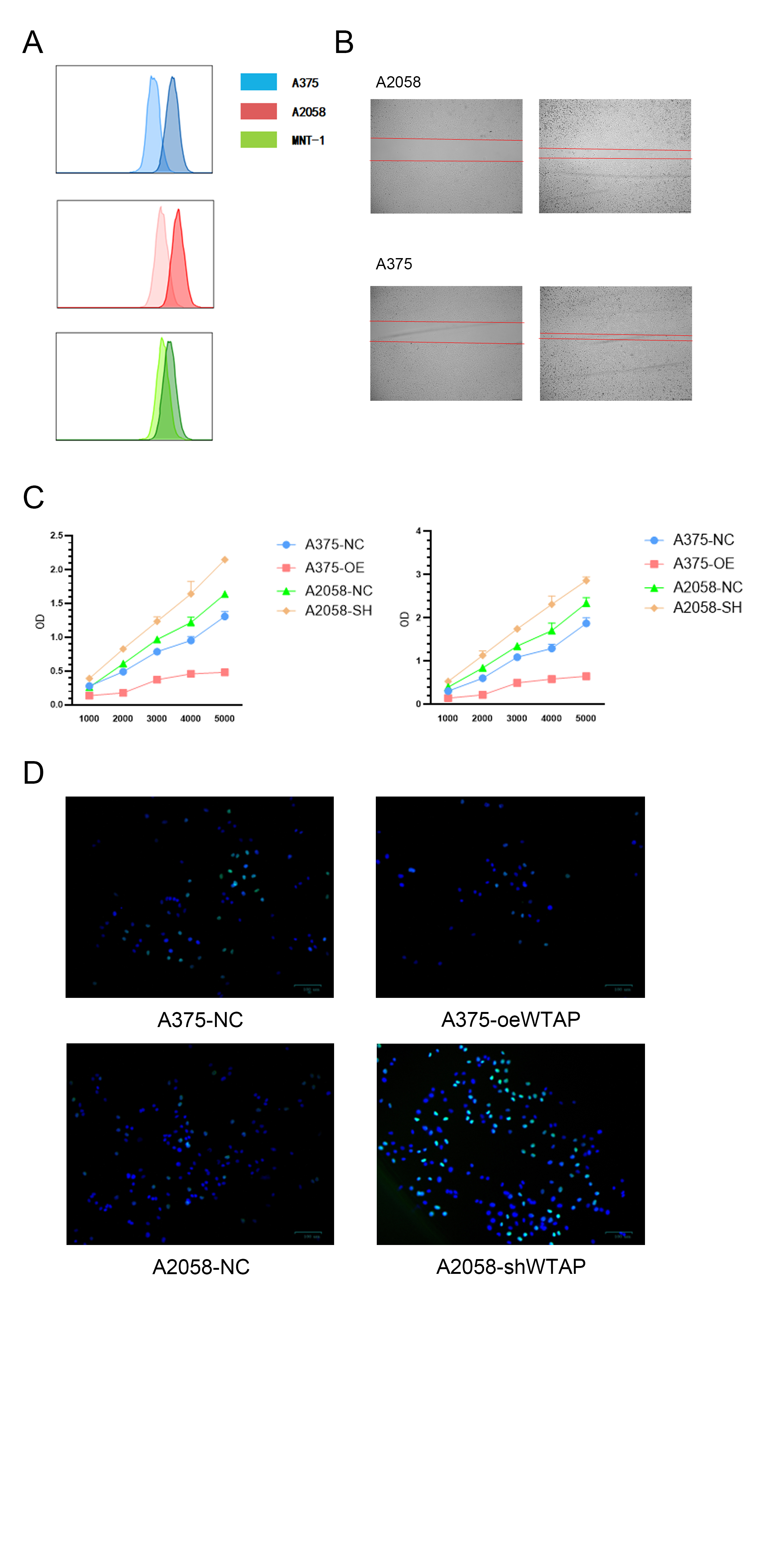

Supplement: Supplementary file 1 [file biomedicines-13-02685-s001.zip › supplementary/Figure S1 revised.jpg]

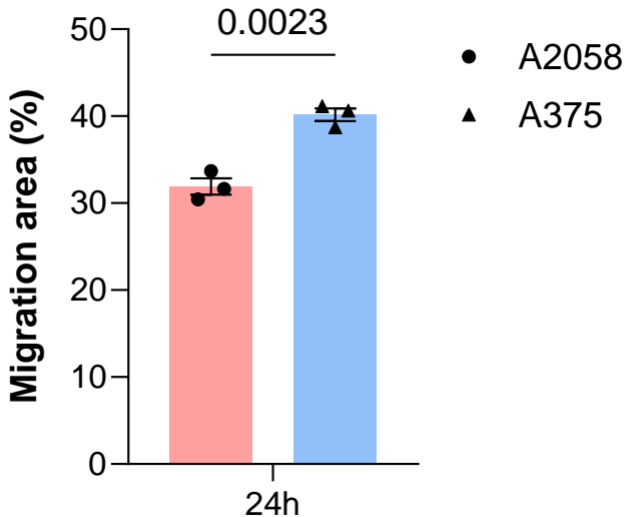

Supplement: Supplementary file 1 [file biomedicines-13-02685-s001.zip › supplementary/Figure S1B_Migration area.pdf]

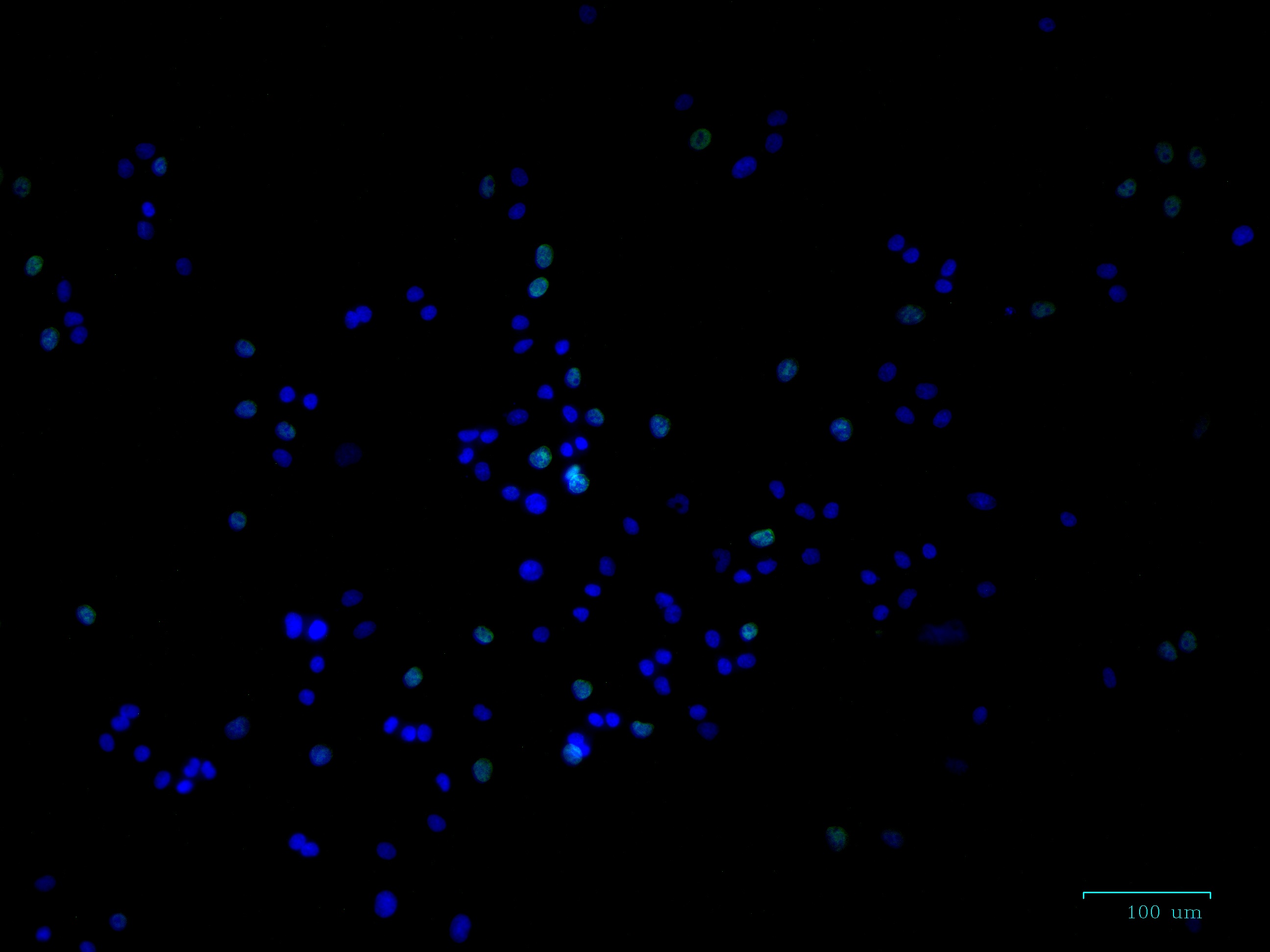

Supplement: Supplementary file 1 [file biomedicines-13-02685-s001.zip › supplementary/Figure S1D raw file/A2058-NC.jpg]

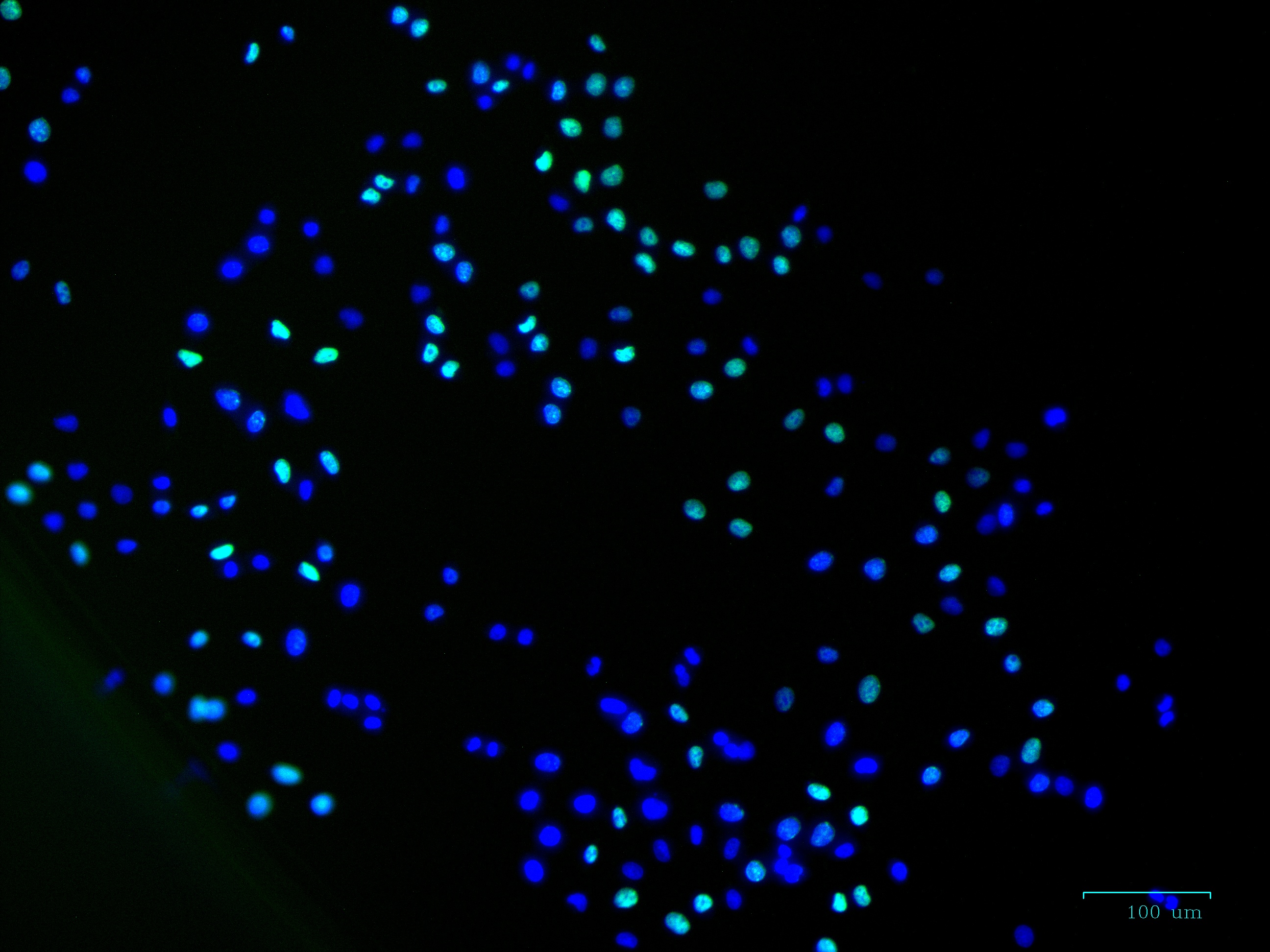

Supplement: Supplementary file 1 [file biomedicines-13-02685-s001.zip › supplementary/Figure S1D raw file/A2058-oeWTAP.jpg]

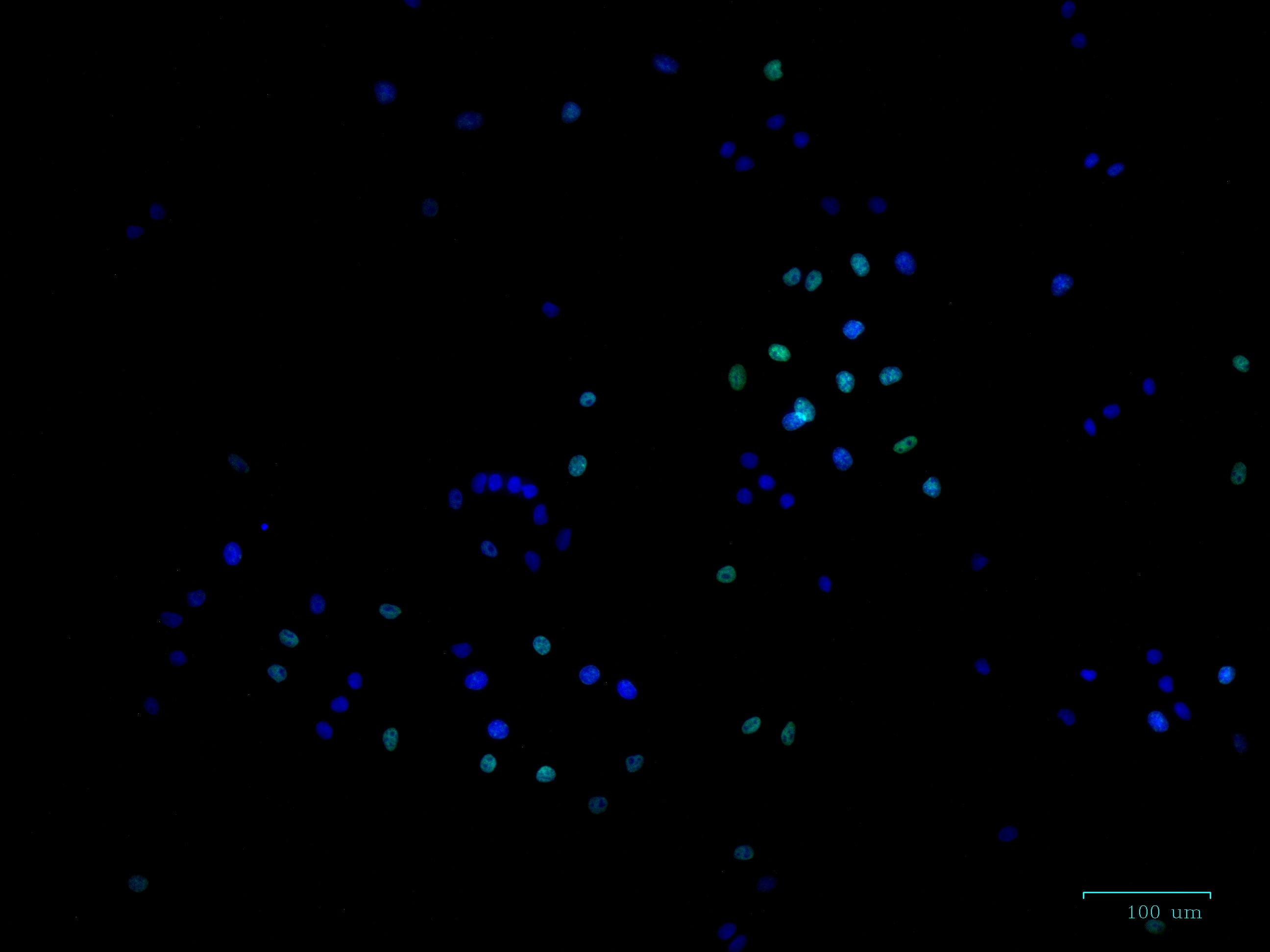

Supplement: Supplementary file 1 [file biomedicines-13-02685-s001.zip › supplementary/Figure S1D raw file/A375-NC.jpg]

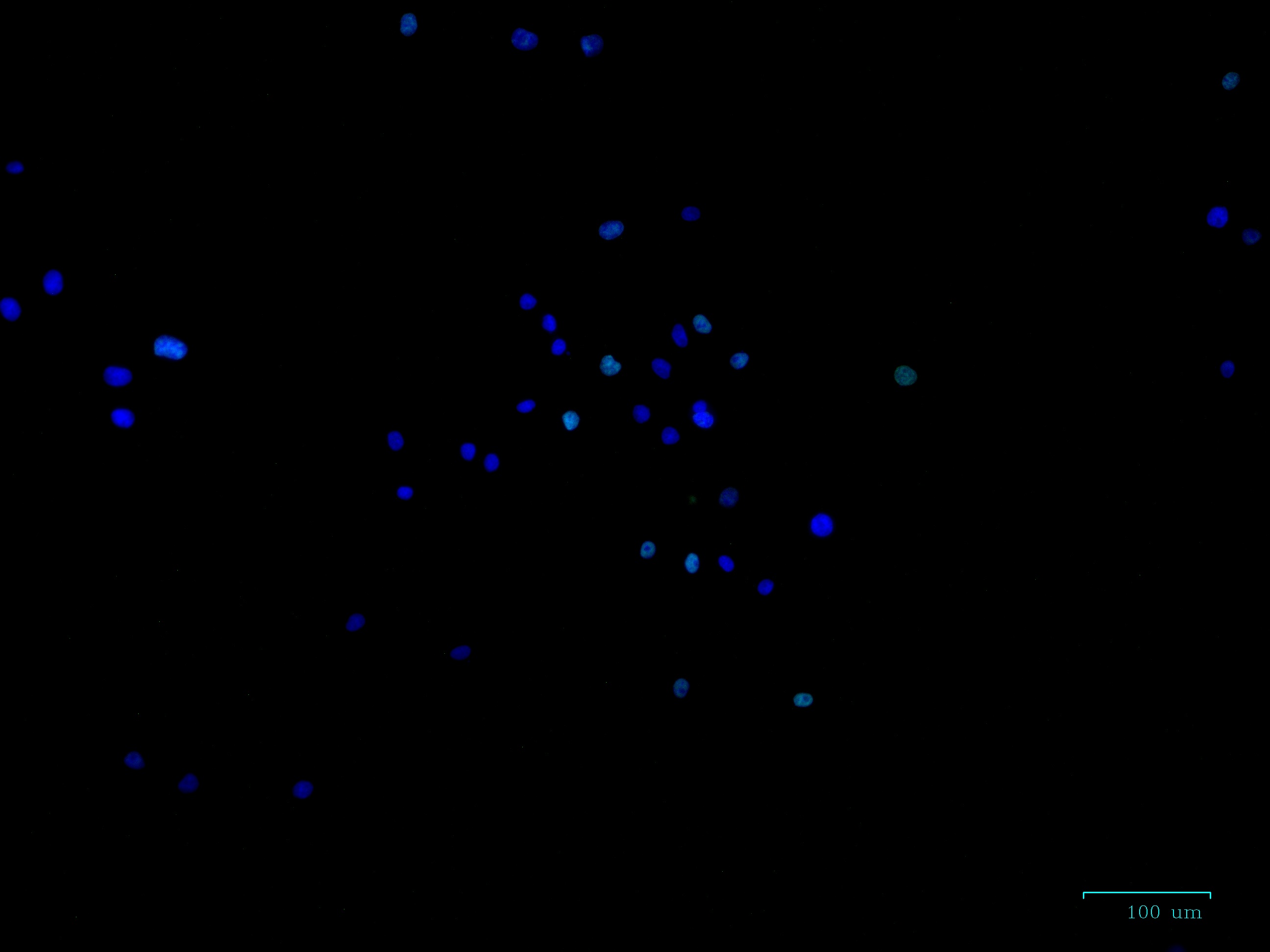

Supplement: Supplementary file 1 [file biomedicines-13-02685-s001.zip › supplementary/Figure S1D raw file/A375-oeWTAP.jpg]

**EdU-positive cells percentage (%)**

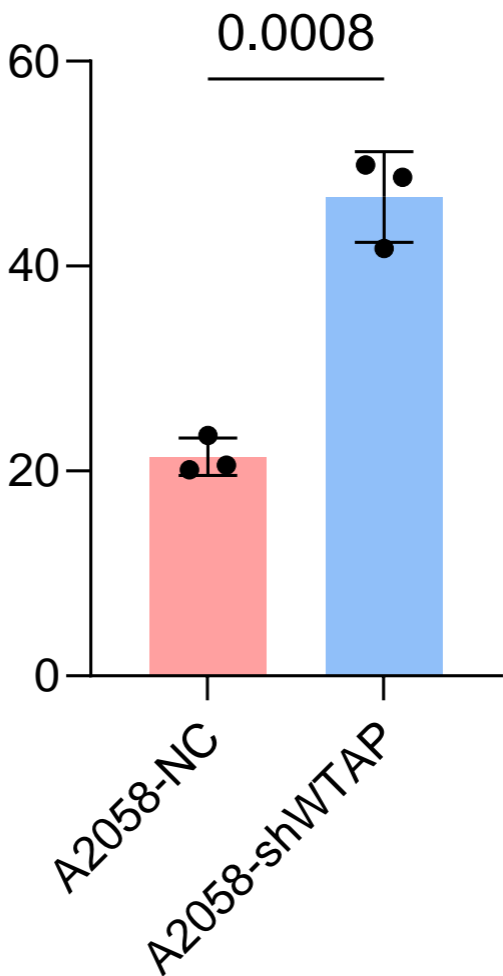

Supplement: Supplementary file 1 [file biomedicines-13-02685-s001.zip › supplementary/Figure S1D_A2058 NC vs shWTAP.pdf]

**EdU-positive cells percentage (%)**

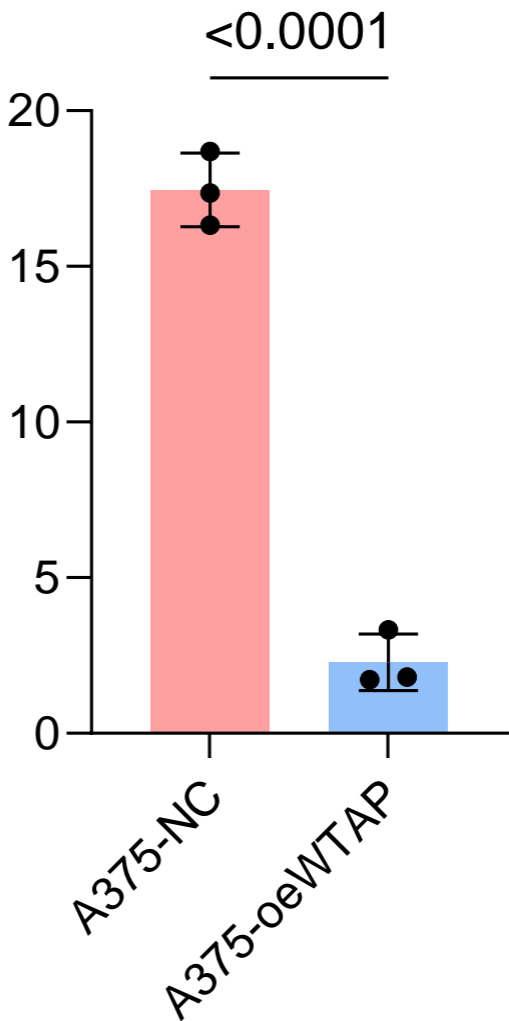

Supplement: Supplementary file 1 [file biomedicines-13-02685-s001.zip › supplementary/Figure S1D_A375 NC vs oeWTAP.pdf]

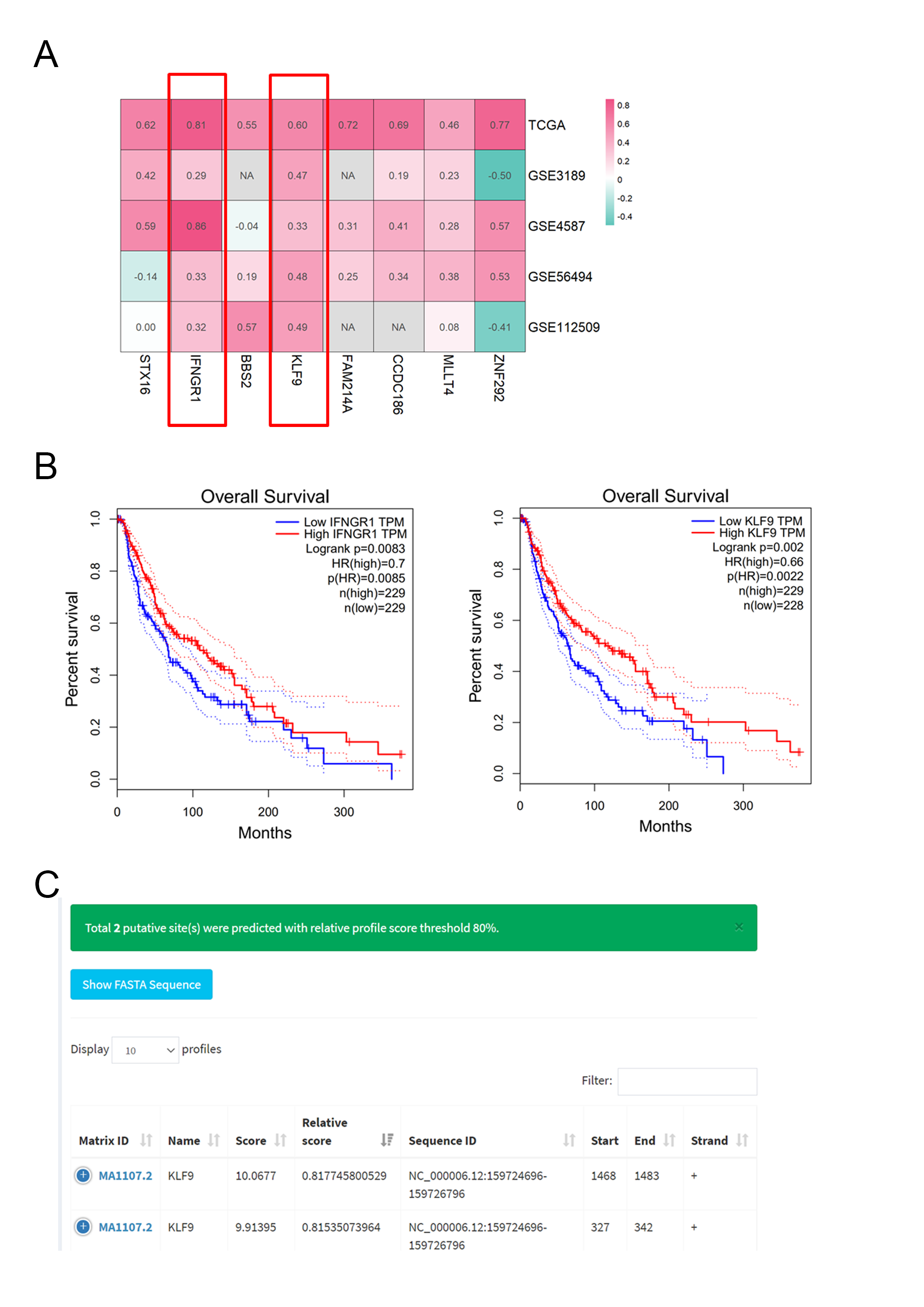

Supplement: Supplementary file 1 [file biomedicines-13-02685-s001.zip › supplementary/Figure S2.png]

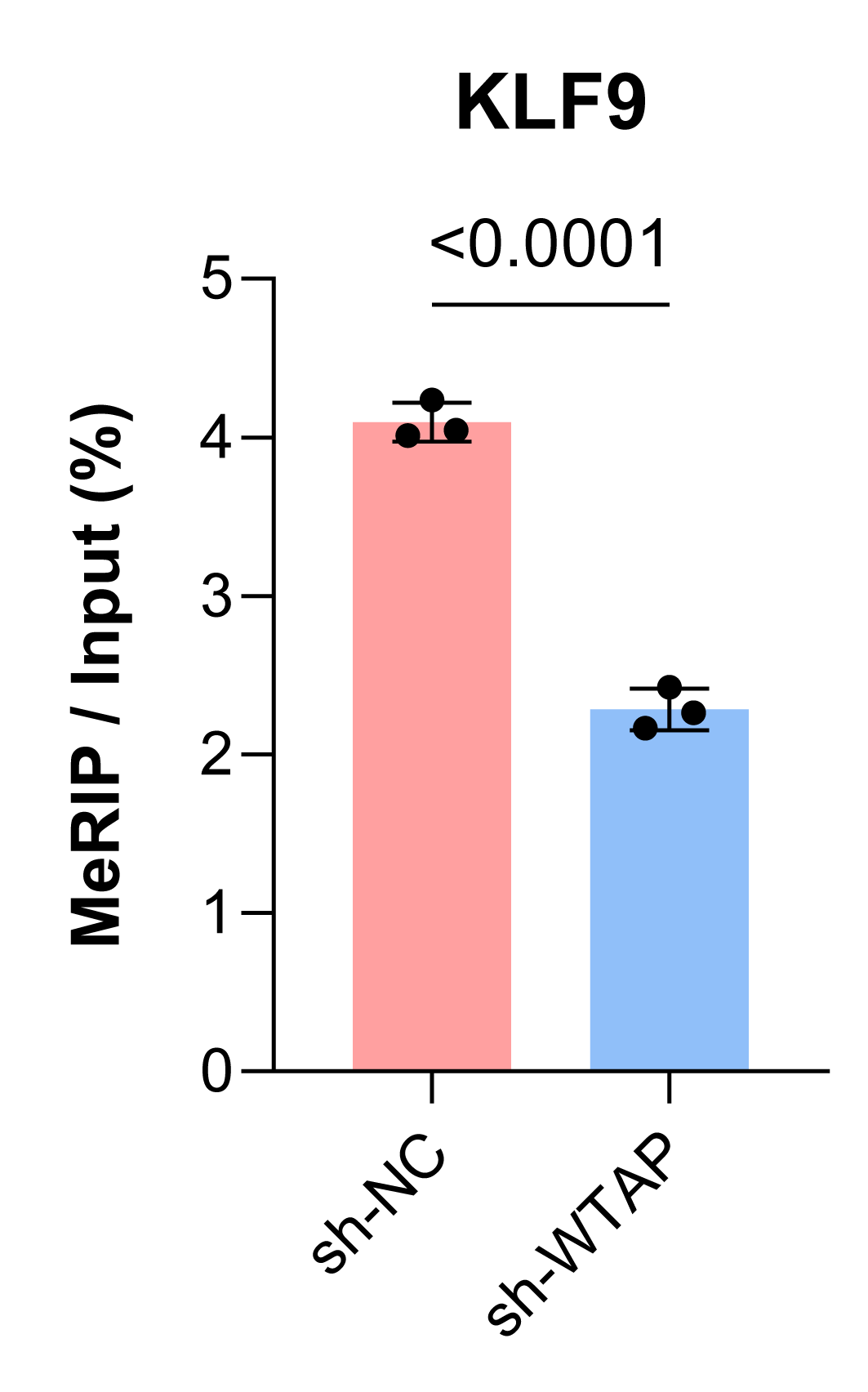

Supplement: Supplementary file 1 [file biomedicines-13-02685-s001.zip › supplementary/Figure S3.tif]

# KLF9

$<0.0001$

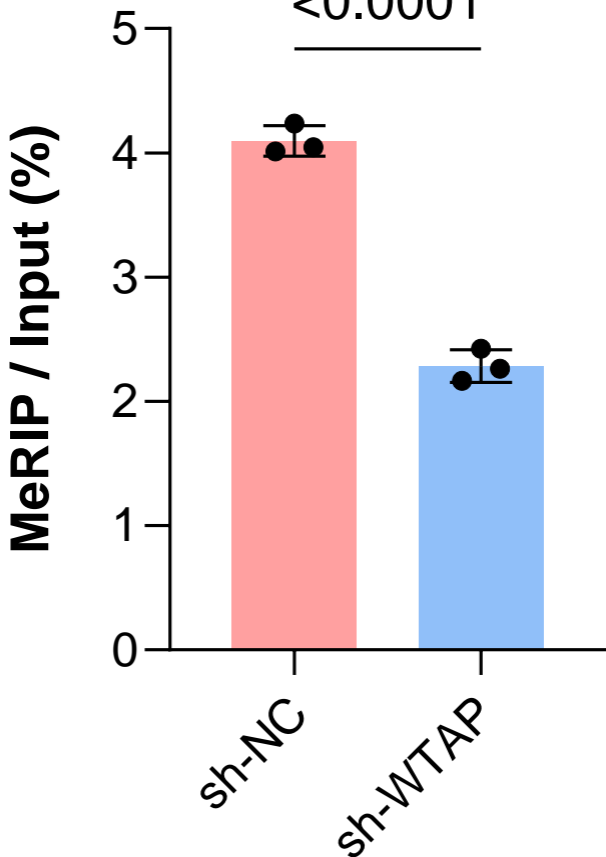

Supplement: Supplementary file 1 [file biomedicines-13-02685-s001.zip › supplementary/Figure S3_MeRIP-qPCR.pdf]
